# Editorial: Perspectives and Theories of Social Innovation for Ageing Population

**DOI:** 10.3389/fsoc.2020.00006

**Published:** 2020-02-25

**Authors:** Andrzej Klimczuk, Łukasz Tomczyk

**Affiliations:** ^1^Warsaw School of Economics, Warsaw, Poland; ^2^Institute of Educational Sciences, Faculty of Education, Pedagogical University of Kraków, Kraków, Poland

**Keywords:** ageing, social gerontology, social innovation, caregivers, age-friendly, public health, healthcare

## Overview

Gerontology together with its subfields, such as social gerontology (sociology of ageing), geragogy, educational gerontology, political gerontology, environmental gerontology, and financial gerontology, is still a relatively new academic discipline that is currently intensively developing, expanding research fields and combining various theoretical and practical perspectives. The interdisciplinarity, transdisciplinarity, and multidisciplinarity of research on ageing and old age, despite its vast thematic, methodological and theoretical diversity, have a common denominator, which is the focus of research work on improving the quality of life of older people (see Fabiś et al., 2015). It is the concern for the components of quality of life such as welfare and well-being as well as focus on learning about phenomena conditioning successful ageing that has become motivators and premises hidden or visible in many texts included in the Research Topic “Perspectives and Theories of Social Innovation for Ageing Population.”

The Research Topic that we are presenting to our readers is unique not only because of its size but above all because of its novelty and social involvement, visible in the content of individual chapters. The presented collection includes 17 articles prepared in total by 76 authors from the following countries: China, Finland, Germany, Ghana, Hungary, Ireland, Israel, Italy, Japan, the Netherlands, Poland, Portugal, Slovenia, Spain, Sweden, the United Kingdom, and the United States. Two journals were combined with this Research Topic: “Frontiers in Public Health” and “Frontiers in Sociology.” The presented Research Topic contains seven types of articles covering: two community case studies (Brown et al.; Pinzón-Pulido et al.), eight original research articles (Berde; Bjursell; Dovie; Senior; Spinelli et al.; Stypińska et al.; Wanka; Zhang and Yang), two perspective articles (Aoo et al.; Piel and Robra), one hypothesis and theory paper (Toczyski et al.), one policy and practice review (Tziraki-Segal et al.), one methods article (Ramovš et al.), and two book reviews (Cieśla; Leszko).

The rationale to start work on this set of texts was the desire to continue and deepen the research analyses of the editors of this set, which concern the development of social innovations for the ageing population as well as changes of public policy on ageing (the ageing policy) (see Klimczuk, 2015, 2017; Tomczyk and Klimczuk, 2015). This Research Topic deals with topics covering issues such as social learning, intergenerational transmission, senior entrepreneurship, creative content creation by older adults, care services, raising the independence of older people in their living environments, dementia challenges, the image of the older generation in local social policies, new trends in qualitative research on old age, strategies for dealing with chronic diseases, the use of digital tools in health education, the silver economy/longevity economy, age-friendly environments, the diversity of structures and social services, preparation for old age, and food safety. The wide variety of submitted texts shows several selected and, according to the authors, current challenges that contemporary seniors have to face. The articles comprising this Research Topic are organized according to five themes.

## Theme I: Critical Evaluation of the Social Image of Older People

Aoo et al. balance in their study between the historical perception of older people in authority status and the current perception of many seniors as beneficiaries of effective social policies. Japanese researchers are engaged in a fair discussion that shows an attempt to create a balance between modern seniors' support systems and the historical role of a sage or mentor. A further study by Wankaine addresses the universal issue for all people who are leaving their work and retiring. A researcher from Germany showed various connotations regarding the retirement period and related consequences in the individual dimension. This article is part of a broad discussion on the changes that occur under the influence of cessation of professional activity. This is also one of the priority research areas for the economies of rapidly ageing countries. In the next chapter Piel and Robra, using the achievements of critical gerontology (see Estes and Phillipson, 2007; Moody and Sasser, 2018) ask about the type and functioning of people in late adulthood in the area of dominant models of intercourse with each other. Researchers question the common images of heterosexual couples and refer in their analyses to the deconstruction of the current model of close interpersonal relationships in old age. The presented studies may also be used to eliminate negative stereotypes regarding old age.

## Theme II: Innovation and Quality of Life in Ageing Societies

The team of Spinelli et al. presents a multitude of threads regarding individual and broader conditions to improve the quality of life. The narrative by British scholars shows new ways of conducting action research through living labs that serve for testing innovative social tools and public services. According to the authors of this chapter, innovation is not only to be an exemplification of social policies focused on the well-being of the individual but also to strengthen the broadly understood economy. The article by Senior is not only related to an earlier study by further focusing on examples from the United Kingdom, but also by highlighting the importance of following non-standard paths in the search for alternatives, new methods, and cross-sectoral cooperation in solving health problems of older people. The study by Bjursell is focused on the area of new methods for discovering limiting and stimulating factors. The Swedish scholar refers here to the popular and well-known concept of the social research methodology, which is based on biographical techniques (cf. Fabiś et al., 2017). Berde, representing Hungarian research institutions, contributes to the discussion on the topic of age discrimination and ageism as well as shows critical and constructive examples of such issue which are taking into account the realities of the Central and Eastern Europe. The text focuses on one of the many predictors of age discrimination and links to the opposite axis of the issue, which is the removal of marginalizing factors in both the workplace and leisure activities (see Veteška, 2016). This multithreaded and multifaceted view on issues of innovation and quality of life ends with two reviews of publications on contemporary trends related to the population ageing. Cieśla the topic of age-friendly cities and communities. While Leszko at the descriptions of the concept of the silver economy/longevity economy. Both scholars discuss the advantages and disadvantages of policy ideas that are gradually emerging around the world in various forms, along with seeking positive responses to the challenges of the ageing population.

## Theme III: Innovation in the Context of Supporting Active Ageing and Healthy Ageing

The next text in this Research Topic for the first insight stands out due to the synergy of cooperation between thirty authors from eight countries who have developed a common position on the issue of health promotion by fostering the development of a culture of pro-health behavior. Tziraki-Segal et al. correctly note that health is a salutogenic and direct element of lifelong education as well as is closely linked to politics, business, and research. In the next article, scholars representing four different research centers (Pinzón-Pulido et al.) refer directly to the previous text regarding issues related to the promotion of healthy lifestyles and co-designing of public health solutions. In their study, Spanish scholars clearly argue the need to strengthen both active ageing and healthy ageing in line with the standards developed by the World Health Organization (WHO) and to improve the exchange and use of best practices. This study is in line with the triangulation of medical outlook with lifelong education that serves and promotes successful ageing as well as healthy ageing (see Mackowicz and Wnek-Gozdek, 2018). Another article written by seven researchers from the United States (Brown et al.) focuses on a very narrow medical area which is support for people with cystic fibrosis in the field of nutritional safety.

## Theme IV: Transformation and Modernization of Care Services

Ramovš et al. present an exemplification of social learning in the perspective of exchange of experience and preparation of staff providing care services addressed to older people. The text brings a new look at the learning process using the accumulated experience of seniors as well as popular gerontological theories which are known for decades. Meanwhile, the next text by Dovie introduces readers to the topic of social policy mechanisms in the field of care services and the health sector. The systemic and individual perspectives on the challenges typical for the Ghanaian society are a pretext to undertake a broader and global debate on the issue of health inequalities and well-being in old age. Despite the seemingly local overtones of Dovie's study, it can also be seen as an example of discussions taking place in many countries struggling with the consequences of an ageing population in the aspect of insufficient efficiency of the healthcare system. Moreover, Zhang and Yang in their chapter, attempted to build a typology of care services practices in China. This is an article familiarizing readers with models of local solutions in the care for older people. The study also stimulates further discussion on the factors of efficient and culturally compatible people-centered care systems in individual societies.

## Theme V: Development of Key Competencies and Creativity of Older People

Stypińska et al. presented a practical discussion on one of the key competences, promoted and defined by the institutions of the European Union. According to German scholars, the senior entrepreneurship could be one of the answers to the shortage of employees and the other consequences of demographic changes for labor markets. The study also shows how important it is to relate the typology of key competencies to opportunities and challenges as well as the accumulated resources of older people. The last chapter of the Research Topic focuses on the example of creative ways to strengthen digital skills (which are also one of the key competencies) among older people. Given the global data on the digital divide (see Tomczyk et al., 2019), this phenomenon requires continuous action to ensure effective digital inclusion. One such example can be seen in an article whose authors (Toczyski et al.) combine crystallized knowledge of older people with issues related to minimizing digital exclusion.

## Conclusion

The research results presented in the articles of this Research Topic allow formulating several hypotheses. Firstly, gerontology is an interdisciplinary field with a high level of innovation potential (Szarota, 2004). Secondly, both new research areas and innovative, practical solutions are determined by the culturally diverse perception of old age and various public interventions in individual countries undergoing the ageing of the population. Thirdly, the measure of social solidarity and maturity is the search for solutions that are characterized by a high level of innovation and pragmatism, aimed not only at children and young people but also at people in late adulthood (see Tomczyk, 2015).

The authors, intentionally selecting the topics presented above, also show the directions in which gerontology could develop. These are both new perspectives in the “soft” areas, e.g., educational, interpersonal relationships, self-improvement, and strengthening of activity, as well as in the “hard” areas when it comes to information and communications technologies (ICTs) and medical, economic, or biological issues. The authors are aware that due to demographic changes and redefinition of views, according to the current of critical gerontology (Formosa, 2005; Mackowicz and Wnek-Gozdek, 2016), new challenges will emerge not only for the individual responsible for their preparation for old age but also before societies of solidarity and innovation. One can risk saying that for the authors of the chapters contained in “Perspectives and Theories of Social Innovation for Ageing Population,” old age appears as a period of life including challenges resulting from deficits and characteristics specific to this development period. At the same time, despite the awareness of the negative consequences associated with the ageing process, the authors constructively express the possibilities inherent in late adulthood, both in individual and collective dimensions. Selected texts can also be read at the level of meta-theoretical assumptions, in which old age appears to be a favorable stage of life in the macro-social perspective, because it contributes to the development of innovation, and thus also to the improvement of the quality of life of all ageing people. Also, a deep reflection on late adulthood strengthens and further develop social theories of ageing including, among others, sociology, economics, public policy, management and organization, social work, and pedagogy.

We present to our readers a Research Topic characterized by a deep and multi-directional discourse on the ageing process and the phenomenon of old age. The study also has universal elements in a global perspective. The challenges and opportunities presented by the authors in a sense create a new quality also in the perspective of the functioning of “global seniors” who, similarly to representatives of other age categories (e.g., global teenagers), despite cultural, geographical, and economic differences face similar biopsychosocial challenges.

## Author Contributions

The lead author of this editorial is ŁT. AK outlined, drafted the editorial, contributed by reviewing and revising the manuscript of editorial and leading editorial work on all manuscripts included in this Research Topic. All authors of papers listed have made a substantial, direct and intellectual contribution to the work, as well as approved their papers for publication.

### Conflict of Interest

The authors declare that the research was conducted in the absence of any commercial or financial relationships that could be construed as a potential conflict of interest.
